# Inflammatory aneurysm of ascending aorta and left anterior descending artery

**DOI:** 10.1186/s13019-022-02006-2

**Published:** 2022-10-01

**Authors:** Amanda Stella, Alexandra Tuluca, Anastasia Arce, Louis Samuels

**Affiliations:** Division of Cardiothoracic Surgery, Department of Surgery, Albert Einstein Medical Center, 5501 Old York Rd, Philadelphia, PA 19141 USA

**Keywords:** Inflammatory aneurysm, Ascending aorta, Aortitis

## Abstract

**Background:**

Inflammatory aortic aneurysms represent less than 5% of ascending aortic aneurysms and are mostly diagnosed intraoperatively. Furthermore, to our knowledge, a case of an accompanying inflammatory LAD aneurysm has not been previously reported.

**Case presentation:**

We report an unusual case of a 42-year-old female with a symptomatic inflammatory aneurysm of the ascending aorta and the LAD. The patient underwent successful resection and replacement of the aneurysm as well as bypass of the LAD.

**Conclusions:**

The underlying etiology of severe inflammatory ascending aortic aneurysms in a previously healthy individual remains unknown. The pre-operative diagnosis is difficult to obtain as it does not have a characteristic appearance on currently available imaging modalities.

## Background

Inflammatory aortic aneurysms are rare and typically located in the infra-renal abdominal aorta. It contributes to less than 5% of abdominal aortic aneurysms [1]. In 1972, Walker et al. was the first to expound on this discrete entity which has significant gross pathologic features including wall thickening with extensive fibrous adhesions [2]. There are distinct histopathologic differences as well including marked destruction of media with heterogenous lymphoplasmacytic infiltrate and rare giant cells. Although they behave like their counterparts in the abdomen, inflammatory aortic aneurysms of the ascending aorta are even more uncommon. In 1994, Connery et al. was the first to report an inflammatory aneurysm in the ascending thoracic aorta; however, this patient died shortly post-operatively [3]. Also, there are no reports of this inflammatory process extending into coronary vessels. We report a successful case of an inflammatory aneurysm of the ascending aorta with comparable inflammation of the left anterior descending coronary artery (LAD) in a young female patient.

## Case presentation

**Fig. 1:**
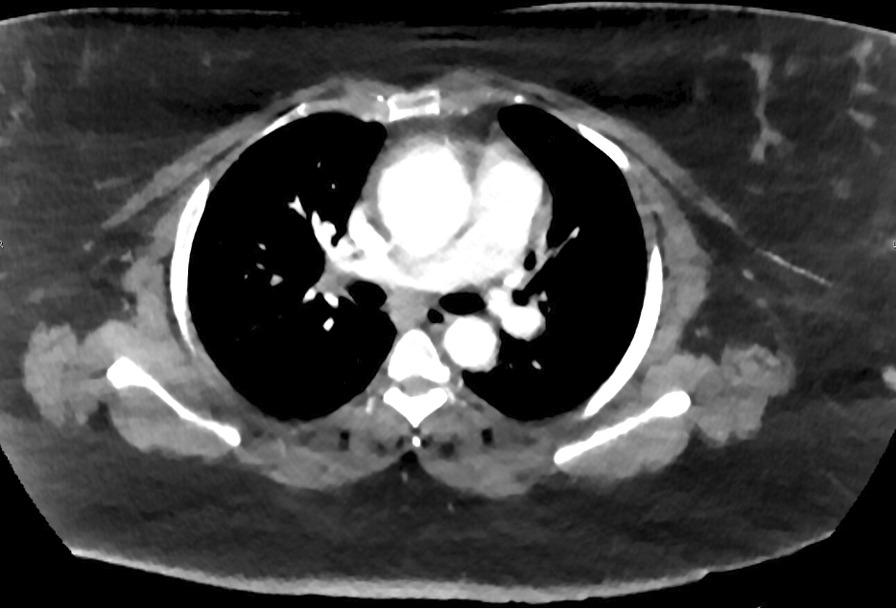
Computed tomogram of an ascending aortic aneurysm with a suspected intramural hematoma

**Fig. 2:**
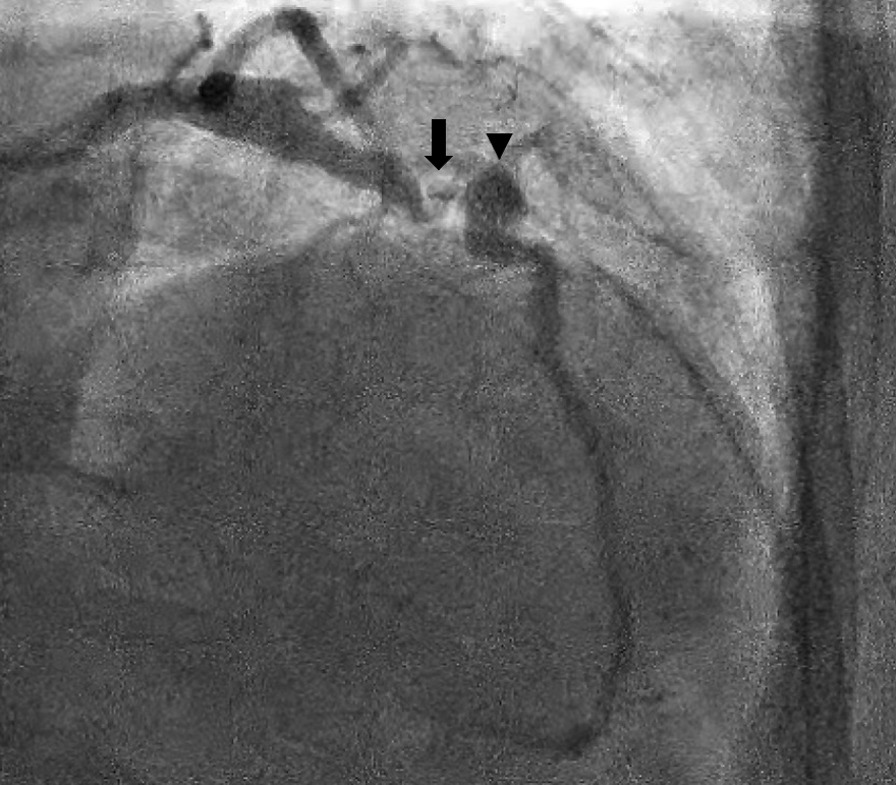
Left heart catherization identifying the left anterior descending coronary artery with 85% stenosis (arrow) followed by a mid-saccular aneurysm (arrowhead)

A 42-year-old female with no significant past medical history presented with worsening shortness of breath and chest pain. She has a significant family history of cardiac disease consisting of coronary artery disease (CAD) and valvular disease in her mother, CAD in her father, and two sisters both with congestive heart failure. She had been admitted 1 month prior for sharp, midsternal chest pain that radiated to the right arm, new onset dyspnea with minimal activity and ankle edema. At that time, a myocardial infarction had been ruled out as the electrocardiogram and troponin was normal three times. A Transthoracic echocardiogram (TTE) showed the aortic root to be 3.5 cm with a normal ejection fraction greater than 55%. A Computed tomography (CT) was performed which identified an aneurysmal dilatation of the mid ascending aorta measuring 5.1 cm with extensive calcifications seen along the LAD and right coronary artery concerning for extensive atherosclerosis. The follow-up visit 1 month later consisted of a TTE that identified a dilated aortic root at 4.1 cm and a CT Angiography that identified a 5.7 cm fusiform aneurysm of the ascending thoracic aorta with a suspected intramural hematoma (Fig. 1). Additionally, Left Heart Catherization identified the LAD to have a mid-saccular aneurysm which was immediately preceded by a severe 85% eccentric lesion and followed by a second 70% stenosis (Fig. 2).

**Fig. 3:**
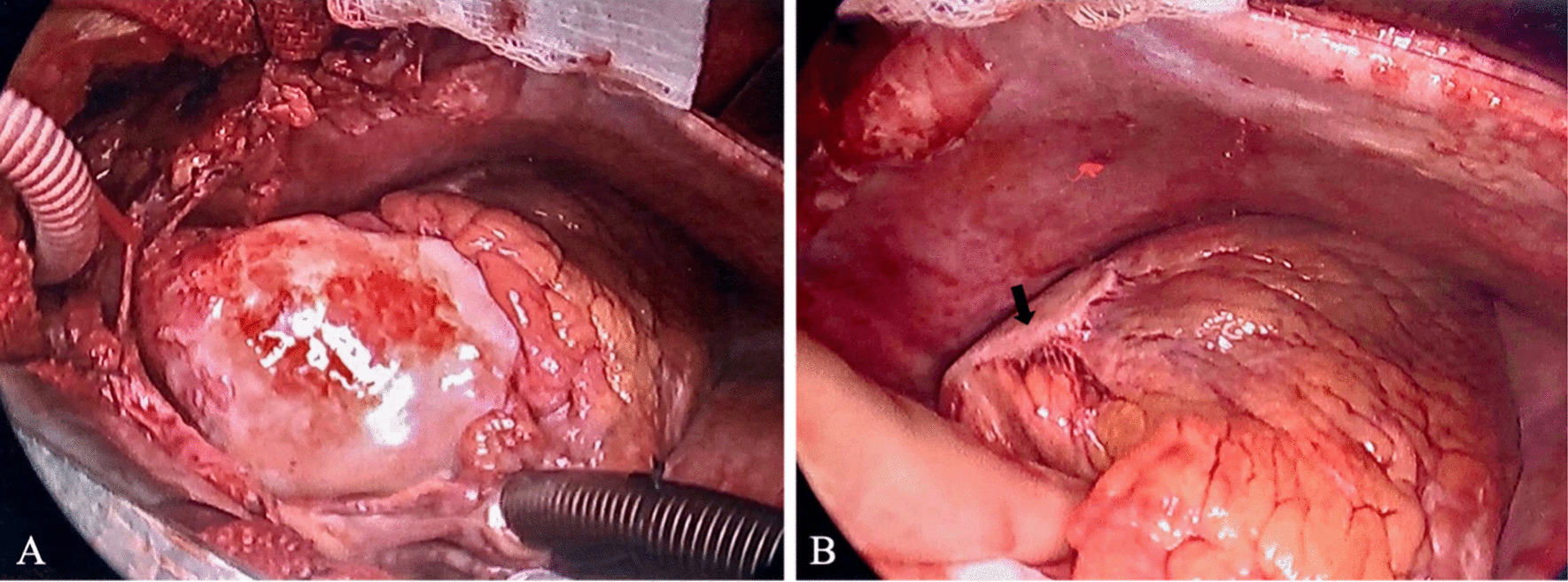
**A** Intraoperative view of the inflammatory ascending aortic aneurysm. **B** Inflammatory plaque overlying the LAD (arrow)

In the operating room, a large inflammatory-appearing aneurysm was identified in the ascending aorta which consisted of a shiny, eggshell appearing glistening capsule. The thick wall appeared pink-yellow and had areas of white-tan fibrosis and calcifications (Fig. 3A). A similar inflammatory, thickened plaque was found overlying the mid-LAD in the area of the presumed aneurysm (Fig. 3B). The ascending aneurysm plaque extended to just proximal to the innominate artery (IA) branchpoint. Therefore, standard aortic cannulation was not possible as there was not enough mobility due to severe tissue inflammation. Interestingly, the patient had a bovine arch anatomy with the left carotid artery (LCA) branching off a common trunk with the IA, which was carefully avoided. A partial occluding clamp was placed on the IA distal to the LCA takeoff. Central cannulation was achieved with a graft to the IA for arterial inflow. Standard cannulation followed with dual-stage venous return through the right atrial appendage. Cerebral protection was achieved by modified deep hypothermic circulatory arrest with antegrade cerebral perfusion via the innominate artery. The ascending aorta and the proximal hemiarch aneurysm were resected and replaced with a 26 mm Gelweave Graft (Terumo Aortic, Sunrise, FL). A coronary artery bypass graft was performed bypassing with LAD with the left internal mammary artery. The patient was extubated shortly after the operation, and the remainder of the postoperative course was uneventful.

**Fig. 4:**
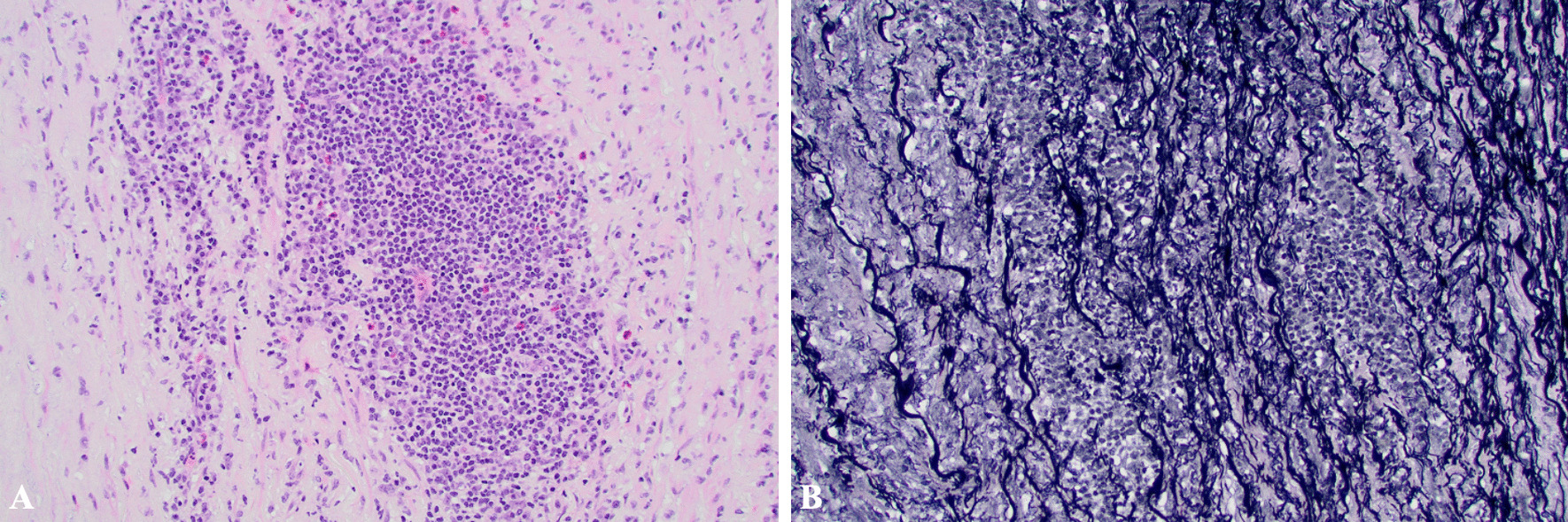
20 × magnification photomicrographs of the aortic media showing **A** lymphoplasmacytic, inflammatory infiltrate with a few eosinophils (H&E stain) and **B** destruction of elastic fibers of the media (elastin stain)

Histologic evaluation of the aneurysm revealed aortitis consisting of lymphoplasmacytic infiltrate with patchy acute inflammation, destruction of the media, and edema with a rare giant cell (Fig. 4A). An elastin stain also demonstrated the destruction of elastin fibers in the area of inflammation (Fig. 4B). Bacterial, fungal, and treponemal stains were negative. Testing for SARS CoV-2 RNA was also negative.

## Discussion and conclusions

Inflammatory aortic aneurysms comprise a minor subset of aortic aneurysms. Connery et al. described the first inflammatory aneurysm of the ascending aortic in 1994 [3]. Since then, only twelve other case studies have been reported, most of which consisted of individuals over the age of 54 or with related inflammatory diseases [1, 4]. Other studies have described incidental findings of aortitis upon retrospective pathology reviews of aortic surgical specimens, however, the severity of inflammation in this case is incomparable [5]. Also, most cases consisted of elderly individuals with related comorbid diseases including giant cell arteritis and other rheumatologic diseases. A severely inflamed ascending aortic aneurysm with the inflammation extending into the LAD in a young female with no related co-morbid diseases is a medical phenomenon that has not been previously documented to our knowledge.

The cause of inflammatory aortic aneurysms is still unclear. Inflammatory diseases of the aorta are thought to be either infectious or non-infectious. Because of the negative bacterial, fungal and treponemal stains, the infectious forms are ruled out in our patient. The role of a viral infection involving SARS CoV-2 was postulated but also found to be noncontributory. The differential diagnosis for a non-infectious aortitis could be due to an autoimmune response against atherosclerosis [6]. Being that this inflammatory process occurred in the occluded LAD as well, it could be postulated that our patient had an autoimmune reaction to the atherosclerotic plaques. Further studies to diagnose an underlying inflammatory, rheumatologic, or autoimmune etiology are required to expound on this phenomenon. Other noninfectious inflammatory diseases have been postulated to be the underlying cause of thoracic aortitis including Takayasu arteritis, granulomatosis with polyangiitis and sarcoidosis [7]. Some studies have reported IgG4-related sclerosing disease is involved in thoracic aortitis [5]. However, some authors contend the elevated IgG4 levels are an incidental finding and don’t contribute to the diagnosis due to the inadequate serologic correlation [7].

Preoperative diagnosis was difficult because CT could not differentiate an inflammatory aneurysm from an intramural hematoma. This drawback is also demonstrated in our literature search where six of the thirteen cases were preoperatively diagnosed with an intramural hematoma. Furthermore, only two of the thirteen cases had accurate preoperative diagnoses of an inflammatory aortic aneurysm [1, 4]. Echocardiogram is also unable to correctly identify this disease due to the echocardiographic similarities of an aortic wall with inflammation, atherosclerosis, and a thrombosed false lumen [1]. The final diagnosis is presumed by the gross appearance during the operation and confirmed by histologic evaluation of the tissue. Therefore, an inflammatory aortic aneurysm should be considered preoperatively when there is an abnormal dilated aortic aneurysm with a thickened wall. At the time of surgery, an inflammatory aneurysm is suspected when there is inflammatory thickening of the aortic wall and excessive adherence to surrounding structures. The histopathologic evaluation of inflammatory aortic aneurysms confirms the diagnosis and typically shows inflammatory lymphoplasmacytic infiltration with destruction of the media.

In summary, we described the case of an inflammatory aneurysm of the ascending aorta with comparable inflammation of the LAD in a young female patient who underwent successful surgical treatment. The underlying etiology of severe inflammatory ascending aortic aneurysms with lymphoplasmacytic infiltration and media destruction without contributory diseases remains uncertain and still open for debate. There is a high surgical mortality (16%) of the inflammatory subtype compared to elective repair of ascending aortic aneurysms [1]. More advanced preoperative imaging is needed to unmask this diagnosis as it is questioned whether this fragile inflammatory aneurysm requires surgery at earlier stages compared to its non-inflammatory counterparts.

## Data Availability

Not applicable.

## Abbreviations

CAD: Coronary artery disease
CT: Computed tomography
IA: Innominate artery
LAD: Left anterior descending coronary artery
LCA: Left carotid artery
TTE: Transthoracic echocardiogram
